# Genetic Modifiers of Neurofibromatosis Type 1-Associated Café-au-Lait Macule Count Identified Using Multi-platform Analysis

**DOI:** 10.1371/journal.pgen.1004575

**Published:** 2014-10-16

**Authors:** Alexander Pemov, Heejong Sung, Paula L. Hyland, Jennifer L. Sloan, Sarah L. Ruppert, Andrea M. Baldwin, Joseph F. Boland, Sara E. Bass, Hyo Jung Lee, Kristine M. Jones, Xijun Zhang, NISC Comparative Sequencing Program, James C. Mullikin, Brigitte C. Widemann, Alexander F. Wilson, Douglas R. Stewart

**Affiliations:** 1 Clinical Genetics Branch, Division of Cancer Epidemiology and Genetics, National Cancer Institute, National Institutes of Health, Rockville, Maryland, United States of America; 2 Genometrics Section, Computational and Statistical Genomics Branch, National Human Genome Research Institute, National Institutes of Health, Baltimore, Maryland, United States of America; 3 Genetic Epidemiology Branch, Division of Cancer Epidemiology and Genetics, National Cancer Institute, National Institutes of Health, Rockville, Maryland, United States of America; 4 Genetic Disease Research Branch, National Human Genome Research Institute, National Institutes of Health, Bethesda, Maryland, United States of America; 5 Pediatric Oncology Branch, National Cancer Institute, National Institutes of Health, Bethesda, Maryland, United States of America; 6 Cancer Genomics Research Laboratory, Division of Cancer Epidemiology and Genetics, National Cancer Institute, National Institutes of Health, Rockville, Maryland, United States of America; 7 NIH Intramural Sequencing Center, National Human Genome Research Institute, Rockville, Maryland, United States of America; Stanford University School of Medicine, United States of America

## Abstract

Neurofibromatosis type 1 (NF1) is an autosomal dominant, monogenic disorder of dysregulated neurocutaneous tissue growth. Pleiotropy, variable expressivity and few *NF1* genotype-phenotype correlates limit clinical prognostication in NF1. Phenotype complexity in NF1 is hypothesized to derive in part from genetic modifiers unlinked to the *NF1* locus. In this study, we hypothesized that normal variation in germline gene expression confers risk for certain phenotypes in NF1. In a set of 79 individuals with NF1, we examined the association between gene expression in lymphoblastoid cell lines with NF1-associated phenotypes and sequenced select genes with significant phenotype/expression correlations. In a discovery cohort of 89 self-reported European-Americans with NF1 we examined the association between germline sequence variants of these genes with café-au-lait macule (CALM) count, a tractable, tumor-like phenotype in NF1. Two correlated, common SNPs (rs4660761 and rs7161) between *DPH2* and *ATP6V0B* were significantly associated with the CALM count. Analysis with tiled regression also identified SNP rs4660761 as significantly associated with CALM count. SNP rs1800934 and 12 rare variants in the mismatch repair gene *MSH6* were also associated with CALM count. Both SNPs rs7161 and rs4660761 (*DPH2* and *ATP6V0B*) were highly significant in a mega-analysis in a combined cohort of 180 self-reported European-Americans; SNP rs1800934 (*MSH6*) was near-significant in a meta-analysis assuming dominant effect of the minor allele. SNP rs4660761 is predicted to regulate *ATP6V0B*, a gene associated with melanosome biology. Individuals with homozygous mutations in *MSH6* can develop an NF1-like phenotype, including multiple CALMs. Through a multi-platform approach, we identified variants that influence NF1 CALM count.

## Author Summary

Neurofibromatosis type 1 (NF1) is a relatively common genetic disease that increases the chance to develop a variety of benign and malignant tumors. People with NF1 also typically feature a large number of birthmarks called café-au-lait macules. It is difficult to predict severity or specific problems in NF1. We sought to identify genes (other than *NF1*, the gene that causes the disease) that influence severity in NF1. We determined the number of café-au-lait macules in two groups of people with NF1. We measured the gene expression of about 10,000 genes in the cultured white blood cells from one group of people. We then sequenced a group of genes whose expression level was increased in people with higher numbers of café-au-lait macules. In the first group, we found common variants in genes *MSH6* and near *DPH2* and *ATP6V0B* that were significantly associated with the number of café-au-lait macules. Some of these variants were close to significant in the second group of people. The two variants near *DPH2* and *ATP6V0B* were very significant when analysed in both groups combined. Our work is among the first to identify genetic variants that influence the severity of NF1.

## Introduction

Neurofibromatosis type 1 (NF1) is a common, monogenic disorder of dysregulated tissue growth that is caused by mutations in the tumor suppressor gene *NF1* (chromosome 17q11.2). Neurofibromas, soft fleshy tumors, are the hallmark lesion of NF1; affected individuals may have dozens to thousands of neurofibromas. Other clinical features include multiple café-au-lait macules (CALM) on the skin, axillary and groin freckling, benign tumor-like lesions of the iris (Lisch nodules), scoliosis, enlarged head circumference and learning disabilities. Individuals with NF1 are also susceptible to variety of other benign and malignant tumors [1].

Although the allele responsible for NF1 is inherited in an autosomal dominant pattern, the NF1 phenotype is complex because of variable expressivity, pleiotropy and limited *NF1* genotype-phenotype correlates [2], [3]. The inability to predict the severity of phenotype in NF1 has important clinical consequences and essentially precludes prognostication regarding disease severity even among family members who share an identical *NF1* mutation. “Simple” monogenic disorders like NF1 are often more complicated than expected, and thus comprise a potential model for studying complex traits [4], [5], a term usually reserved for disorders like diabetes, which cluster in families but typically are not due to single-gene Mendelian inheritance. The phenotypic complexity of NF1 likely is multifactorial, including epigenetic phenomena, stochastic events and heritable elements such as genetic modifiers [6].

There is experimental and clinical evidence that genetic modifiers explain a major fraction of phenotypic variation in NF1. In mice, specific loci responsible for susceptibility to astrocytoma/glioblastoma in male mice (*Arlm1*) [7], resistance to spinal cord astrocytoma in mice (*Scram1*) [8], and murine peripheral nerve sheath tumors (*Nstr1* and *Nstr2*) [9] have been identified [9], [10]. In one study in humans, correlation between CALM count and cutaneous neurofibroma burden was highest among monozygotic twins and decreased successively among first- and second-degree relatives. Furthermore, four of the five binary traits studied (presence/absence of plexiform neurofibromas, optic pathway gliomas, scoliosis, epilepsy and need for remedial education) also showed significant familial clustering [11]. Szudek *et al.* observed similar patterns of intra-familial phenotype correlation that suggested a role for genetic factors [12]. An analysis of NF1 phenotype presence and severity in a large French cohort found patterns of familial correlations that indicated a strong genetic component, with no apparent influence of the normal (non-mutated) germline *NF1* allele [13].

Only a few genes and loci influencing the NF1 phenotype have been found to date. In a pedigree with both NF1 and congenital megacolon, only members with both the paternally derived *GDNF* R93W allele and maternally inherited *NF1* mutation had megacolon [14]. In a study of neurofibroma burden in NF1, evidence of a higher rate of DNA mismatch repair (MMR) gene *MSH2* (but not other MMR genes *MLH1*, *MSH6* or *PMS2*) promoter methylation was observed in NF1 cases compared with controls. Among NF1 patients with higher tumor count, statistically significant enhanced methylation of two (of six) CpG islands in *MSH2* was observed in 79 NF1 patients, versus 39 controls [15]. Beyond the MMR pathway, the noncoding RNA gene *ANRIL* is transcribed in the antisense orientation to *CDKN2A* and *CDKN2B* genes and influences their expression. *ANRIL* was deleted in six of 22 plexiform neurofibromas, as detected by genome-wide array comparative genomic hybridization. Using a family-based association test, a single SNP (rs2151280) in *ANRIL* was significantly associated with the number of plexiform neurofibromas in a cohort of 740 NF1 patients [16], but not in a cohort of 29 individuals with a microdeletion of *NF1* [17].

To identify genetic modifiers in NF1, we recruited and quantitatively phenotyped two cohorts of individuals with NF1. We used the principles of the genetics of gene expression to develop a screen for candidate genes [18]–[20]. We performed the test of association between transcript abundance (as determined by microarray) and variation in human NF1 quantitative phenotypic severity by simple linear regression to identify candidate loci that may modify quantitative traits in NF1. Also known as “genetical genomics” [21] or expression quantitative trait loci (eQTL) mapping, this approach has been successful in elucidating mechanism and causal genes in animal models [22]–[24] and human disease [25]–[30]. Large effect size and widespread prevalence (especially *cis*-acting variation) in the genome makes eQTL mapping an appealing approach, especially in small studies [31]. Thus, we hypothesized that *normal* variation in germline gene expression confers risk for certain clinical phenotypes in an individual haploinsufficient for *NF1*. Select variants were then genotyped in a validation cohort. We studied gene expression in lymphoblastoid cell lines (LCLs). The use of phenotype-specific tissues (*e.g.*, melanocytes or Schwann cells) in a large study is impractical and we used LCLs as a surrogate tissue. There are no studies comparing the degree of expression overlap between LCLs (Epstein-Barr virus-transformed lymphocytes) with melanocytes or Schwann cells. LCLs share 30% of eQTLs with skin and fat; other studies estimate cis-eQTL overlap between blood and fat to be ∼50% [31].

The selection of which phenotype to study is a key consideration in modifier studies. In NF1, many phenotypic features (*e.g.*, neurofibroma burden) are time-dependent and thus comparisons between groups must take age into account. Although we measured a variety of phenotypic features, in this study we focused on CALM count since it is easily quantified and the complement of CALM is typically stable after early childhood. CALM count shows significant familial aggregation and a pattern of familial correlation that suggest a strong genetic component independent from the influence of the germline *NF1* mutation [13]. Finally, CALM are “tumor-like” in that they follow the Knudsen two-hit hypothesis: melanocytes in these lesions acquire a second somatic mutation in *NF1* [32]. Thus, genes that modify CALM count may also plausibly modify tumor burden.

## Results

### Demographics and quantitative phenotypes of study participants

Table 1 summarizes the demographic and phenotypic data from the datasets collected in the study. The 99 NF1 individual (“DISC”) set included 70 of the 79 individuals used for expression regression (“EXPR”) plus an additional 29 participants.

**Table 1:** Demographic and phenotypic characteristics of expression, discovery, validation and combined groups.

Demographic Feature	EXPR (n = 79)	DISC (n = 99)	REP1 (n = 33)	REP2 (n = 81)	Combined (n = 213)
Age (Mean±SD) (years)	36.41±13.85	36.46±13.81	38.06±12.65	15.18±7.70	28.68±15.70
Gender (Male/Female/unknown)	33/46/0	39/60/0	15/18/0	45/35/1	99/113/1
Race (Caucasian/non-Caucasian)	71/8	91/8	30/3	62/19	183/30
NF1 inheritance (*De novo*/Familial/Unknown)	33/38/8	42/45/12	21/10/2	NA	NA

Phenotypic Feature: Mean±SD (n)	EXPR (n = 79)	DISC (n = 99)	REP1 (n = 33)	REP2 (n = 81)	Combined (n = 213)
Height (centimeters)	164.09±8.32 (78)	163.93±9.09 (97)	165.12±7.40 (32)	148.28±21.79 (79)	158.17±16.91 (208)
Head circumference (centimeters)	57.29±2.27 (78)	57.33±2.33 (94)	57.63±1.87 (31)	56.80±3.51 (63)	57.20±2.73 (188)
Total number of CALM	20.4±11.99 (75)	21.71±12.48 (94)	17.94±10.34 (32)	20.29±10.60 (80)	20.57±11.48 (206)
Cutaneous neurofibroma burden	138.62±189.45 (77)	116.48±174.43 (95)	160.77±226.78 (31)	NA	NA
Lisch nodule count	38.73±53.06 (59)	35.23±48.46 (65)	44.67±58.98 (12)	NA	NA
Cherry hemangioma count	8.15±12.34 (65)	8.05±12.39 (81)	6.48±11.76 (29)	NA	NA

### Linear regression of NF1 quantitative phenotypes on gene expression

We sought to identify genetic modifiers of NF1 by test of association by simple linear regression between variation in quantitative phenotype severity and the expression level of each transcript (among ∼10,000 transcripts expressed in the LCLs). After filtering for the false discovery rate (FDR) <0.30, range of expression level >2, or >6, and biological significance, we identified candidate transcript-phenotype pairs for 80 genes (Table S1: “Set of 80”).

### Quantitative PCR verification of putative candidate modifier genes

We chose 21 genes for verification by measuring their expression with quantitative real-time PCR, using the original set of RNA samples (Table S1: “Set of 21”). We chose genes either by the significance of their association with NF1 phenotypes in the original screen or their biological plausibility. Seven of the 21 transcripts (33%) remained significantly associated with phenotype severity (nominal *p* values<0.05) (Table S1: “Verified 7”; Table S2 and Figure S1A–H). The verified genes included *MED21* and *MSH6* (CALM); *NMT2* and *TMEM109* (Lisch nodules); *FHL2*, *RAB11FIP1* and *PREB* (height).

### Identification of variants associated with CALM count in individuals with NF1

We focused on candidate genes influencing the CALM phenotype only, given its clinical tractability and tumor-like biology. Thus, we identified the coding and limited intronic nucleotide sequence of the following genes in germline DNA: *MSH6*, *MSH2*, *MLH1*, *MED21* and *DPH2*. We sequenced *MSH6* and *MED21* genes because of their highly significant association with CALM count in both microarray and qPCR experiments, and because germline mutations in *MSH6* have been associated with development of café-au-lait macules in non-NF1 patients. We included *MSH2* and *MLH1* because their protein products are known to associate with *MSH6* in functional MMR complexes. Moreover, germline mutations in *MSH2* and *MLH1* have been linked to an NF1-like clinical phenotype with multiple CALM [33]–[35]. Despite of the fact that *DPH2* qPCR did not confirm association of the gene with CALM phenotype, the gene was included in the sequencing phase of the analysis because of its biological function (see Discussion).

By sequencing these five genes in the DISC sample set and performing simple linear (Table 2 and Table S3) and tiled regression (Table 3) analyses using additive, dominant and models with untransformed and log-transformed CALM count, we identified thirteen variants in the genomic regions of *MSH6* and two near *DPH2* and *ATP6V0B* that were significantly associated with CALM count. Significance levels were set at 0.05 for linear and tiled regressions. Each model was evaluated with hotspot-based tile regions. For untransformed CALM, the best-fitted model representing the independent SNVs in TRAP is:
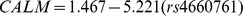
where “rs4660761” represents the number of minor alleles in SNP rs4660761. We did not identify variants in *MED21* or *MSH2* that were significantly associated with CALM count.

**Table 2:** Significance of association of SNVs with CALM count by simple linear regression adjusting for age and sex using self-reported European-American samples.

Model^a^	DISC (n = 89)		REP1 (n = 29)		REP2 (n = 62)		Meta [DISC, REP1, REP2]	Mega [ DISC, REP1, REP2 ]	
	Beta (s.e.)	p-value^b^	Beta (s.e.)	p-value^b^	Beta (s.e.)	p-value^b^	p-value^b^	Beta (s.e.)	p-value^b^
rs7161, chr; 44,211,561 bp in *DPH2*, Minor Allele Frequency from DISC = 0.191									
unt_add	−4.747(2.32)	0.044 *	−1.044(2.67)	0.699	−3.797(2.57)	0.146	0.054	−4.221(1.44)	0.004 **
log_add	−0.055(0.03)	0.080	−0.011(0.04)	0.791	−0.056(0.04)	0.153	0.103	−0.055(0.02)	0.008 **
unt_dom	−5.645(2.55)	0.030 *	−3.014(3.03)	0.329	−4.038(3.16)	0.208	0.024 *	−5.021(1.67)	0.003 **
log_dom	−0.067(0.03)	0.050 *	−0.040(0.05)	0.400	−0.054(0.05)	0.258	0.051	−0.065(0.02)	0.007 **
rs4660761, chr; 44,212,733 bp in *DPH*2, Minor Allele Frequency from DISC = 0.137									
unt_add	−5.318(2.25)	0.020 *	−0.319(2.75)	0.908	−3.127(2.59)	0.233	0.091	−3.978(1.47)	0.007 **
log_add	−0.068(0.03)	0.026 *	0.004(0.04)	0.929	−0.037(0.04)	0.343	0.156	−0.049(0.02)	0.018 **
unt_dom	−6.471(2.52)	0.012 *	−2.239(3.22)	0.493	−2.801(7.96)	0.726	0.108	−5.030(1.68)	0.003 **
log_dom	−0.085(0.03)	0.013 *	−0.022(0.05)	0.651	−0.013(0.12)	0.909	0.269	−0.065(0.02)	0.007 **
rs1800934, chr2; 47,876,485 bp in *MSH6*, Minor Allele Frequency from DISC = 0.199									
unt_add	3.474(2.03)	0.090	−1.248(3.01)	0.682	−2.890(2.49)	0.251	0.126	0.615(1.43)	0.668
log_add	0.059(0.03)	0.027 *	−0.009(0.05)	0.854	−0.054(0.04)	0.142	0.059	0.011(0.02)	0.596
unt_dom	4.212(2.46)	0.091	−1.519(3.74)	0.688	−4.147(3.08)	0.184	0.102	0.560(1.75)	0.750
log_dom	0.076(0.03)	0.020 *	−0.006(0.06)	0.927	−0.068(0.04)	0.135	0.065	0.015(0.02)	0.539

Note:

a) unt: untransformed CALM count; log: log-transformed CALM count; add: additive effect of minor allele; dom: dominant effect of minor allele.

b) *: p-value≤0.05, **: p-value≤0.01.

**Table 3:** Significance of association of SNPs with CALM count by tiled regression of the discovery set.

Position (hg18)	Minor Allele Frequency from DISC	Coding scheme	Model^a^ [TRAP threshold = 0.05]			
			untransformed CALM		log-transformed CALM	
			Beta	p-value^b^	Beta	p-value^b^
(SNP rs4660761) chr1; 44,212,733 bp (*DPH2*)	0.137	the number of minor allele (additive effect of the minor allele) for common SNVs and collapsed variant in hotspot-based regions coded by proportion of the minor allele.	−5.221	0.020 *	−0.067	0.026 *

Note:

a) additive effect of minor allele,

b) *: p-value≤0.05.

### Validation of common SNPs in *MSH6* and near *DPH2* and *ATP6V0B* in an independent sample set

None of the SNPs in *MSH6* (rs1800934) and near *DPH2* and *ATP6V0B* (rs7161 and rs4660761) significant in DISC were significantly associated with CALM count by simple linear regression in REP1 and REP2 (Table 2) at the level of 0.05. In the meta-analysis, SNP rs7161 (*DPH2*) was significant assuming dominant effect of the minor allele using untransformed CALM and SNP rs4660761 (*DPH2*) and SNP rs1800934 (*MSH6*) were marginally significant. In the mega-analysis, SNP rs7161 and SNP rs4660761 (near *DPH2* and *ATP6V0B*) were significant, but not SNP rs1800934 (*MSH6*) (Table 2).

### Functional consequence of variation at SNPs rs466761 and rs7161

**Figure 1:**
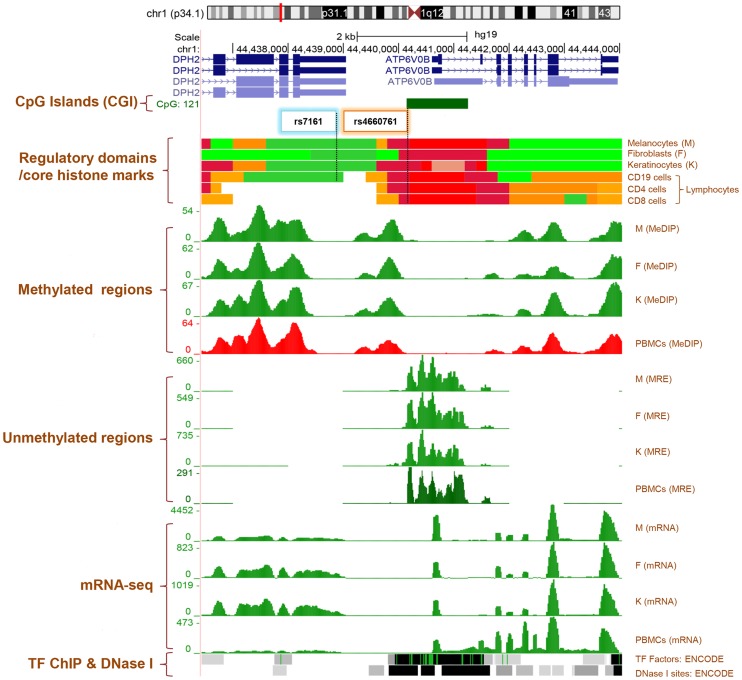
Genome Browser (http://genome.ucsc.edu/) image of *ATPV0B* and *DPH2* gene regions on human assembly hg19 based on NIH Epigenomics Roadmap data and ENCODE data [74], [76]. The promoter CpG islands (CGIs) of *ATPV0B* (CGI: 121) is highlighted by a green filled box. Regulatory domains (chromatin state segmentation using a hidden Markov Model [ChromHMM)] and core histone marks: Crimson, flanking TSS; Red, active transcriptional start site (TSS); Dark Green: transcription elongation/transition; Yellow green: transcription enhancer-like; Orange, active-to-weak enhancer. MeDIP: methylated DNA immunoprecipitation, MRE: methylation-sensitive restriction enzyme sequencing, Melanocytes: normal primary penile foreskin melanocytes (UCSF-UBC-USC and UCSF-UBC), Fibroblasts: normal primary penile foreskin fibroblasts (UCSF-UBC-USC and UCSF-UBC), Keratinocytes: normal primary penile foreskin keratinocytes (UCSF-UBC-USC and UCSF-UBC), PBMCs: peripheral blood mononuclear cells (UCSF-UBC-UCD and UCSF UBC), and Lymphocytes:CD19, CD4 and CD8 cells (NIH Epigenomics Roadmap data). TF: transcription factors ChIP-seq (161 factors) from ENCODE with Factorbook Motifs. DNase I: Open chromatin DNase I hypersensitivity clusters in 125 cell types from ENCODE. SNPs rs4660761 and rs7161 are highlighted by colored boxes. Sources and acknowledgements for the UCSC genome, ENCODE, The NIH ROADMAP databases and extracted tracks http://genome.ucsc.edu/goldenPath/credits.html#human_credits.

Based on Roadmap and ENCODE data, SNP rs4660761 [A/G] is located in an active promoter region and an unmethylated CpG island (CGI) upstream of the gene *ATP6V0B* in normal penile foreskin melanocytes, fibroblasts and keratinocytes (Figure 1). The variant G allele of SNP rs4660761 also creates a CpG dinucleotide within the CGI. The DNA region containing SNP rs4660761 maps to DNase I sites and interacts with a number of proteins in ENCODE cell lines including POL2, and the variant has the potential to alter the DNA binding motifs of BRCA1, YY1 and ZBTB33 proteins (Table S4). SNP rs7161, which is in high correlation with SNP rs4660761 (Pearson correlation coefficient, *ρ* = 0.89), is located in the 3′ UTR region of *DPH2* or 5′ of *ATP6V0B*. SNP rs7161 is reported to locate to an enhancer region with weak H3K4me1 and strong H3K27ac marks in penile foreskin melanocytes using the HaploReg tool (http://www.broadinstitute.org/mammals/haploreg/detail_v2.php?query=&id=rs7161). However, we found no evidence for this enhancer region using Roadmap ChromHMM Primary Core Marks and data from normal melanocytes on the UCSC browser (Figure 1). In K562 and HeLa cells, the DNA region containing SNP rs7161 is strongly enriched for POL2 binding and can also form a chromatin loop structure with the promoter region of the upstream gene *IPO13* (Table S4).

## Discussion

In this study we identified sequence variants that influence CALM count in self-reported European-Americans with NF1. To find genetic modifiers in NF1 subjects we hypothesized that in cells bearing a mutation in the *NF1* gene, normal and genetically determined germline variation in expression level of a potential genetic modifier (other than *NF1* gene itself) will either exacerbate or ameliorate NF1 phenotypes in a quantitative and linear way. We developed a genome-wide screen that regressed transcript expression level against quantitative phenotype to identify transcript-phenotype pairs. We focused primarily on transcripts associated with CALM count, an easily-measured, highly-heritable [13] phenotype; CALM are tumor-like in that they arise from biallelic inactivation of *NF1*. Identification of *MSH6* in the screen also prompted us to sequence *MSH2* and *MLH1*, whose protein products are known to associate with MSH6. Sequencing the *DPH2* locus led to the identification of two SNPs (rs7161 and rs4660761) that were statistically significantly associated, in a variety of models (Tables 2 and 3), with CALM count in NF1 in the discovery (DISC) cohort. In the mega-analysis of all three cohorts (DISC, REP1, REP2), both *DPH2* SNPs in all models were an order of magnitude more significant than in the DISC cohort alone. In addition, the *DPH2* SNP rs4660761 was significant by TRAP analysis in an additive model. Sequencing *MSH6* led to the identification of one SNP (rs1800934) that was statistically significantly associated with CALM count in NF1 in the DISC cohort (assuming dominant effect of the minor allele) and trended to significance in the REP2 cohort. Mega-analysis of all three cohorts for *MSH6* SNP rs1800934 was not significant, although it trended to significance in the meta-analysis. A group of twelve rare (mean MAF = 0.015) *MSH6* SNPs collapsed in hotspot-based regions identified in the DISC cohort were significant in a model coded by the proportion of the minor allele. Given their rarity we did not attempt to validate them in the REP1 or REP2 sets.

The two validated *DPH2* SNPs, rs4660761 and rs7161, are non-coding and reside in the ∼1.5 kb region between the 3′-UTR of *DPH2* and 5′-end of *ATP6V0B*. Genetic variation in *DPH2* and *ATP6V0B* have not previously been associated in any GWAS study with any known human phenotype [36]. The DNA region containing SNP rs4660761 appears to be in the active promoter of the gene *ATP6V0B* in normal melanocytes, keratinocytes and fibroblasts. The region is further enriched for POL2 in K562 cells and the variant G allele of SNP rs4660761 forms the consensus DNA sequence of the binding motif of the transcriptional regulator ZBTB33. A positive relationship between ZBTB33 binding, the absence of DNA methylation, the presence of active promoter marks and gene expression in K562 and GM12878 cells has been reported [37]. Collectively, these data suggest an important function for SNP rs4660761 in the transcriptional regulation of *ATP6V0B*. The SNP rs7161 is upstream of SNP rs4660761, and while these two SNPs are in high correlation (Pearson's correlation of 0.836 (in DISC) and 0.817 (DISC+REP1+REP2)) in our population, SNP rs7161 does not appear to be in a regulatory region in melanocytes, fibroblasts or keratinocytes. We observed higher mRNA levels of *ATP6V0B* in melanocytes compared to fibroblasts, keratinocytes and PBMC cells using Roadmap RNA-sequence data. However, data from neXtProtein suggests that ATP6V0B is only expressed at the protein level in melanocytes (http://www.nextprot.org/db/entry/NX_Q99437/expression).

ATP6V0B is a subunit of the V0 membrane integral domain (or proton-conducting pore) of the vaculolar ATP multi-protein complex (V-ATPase) [38]. V-ATPases are known for their role in H^+^ transport in which they are important for intracellular and extracellular acidification events, protein transport and membrane fusion [39], [40]. Importantly, V-ATPase function is essential for melanosome biogenesis [41]. In fact, melanosomes are acidic organelles where low luminal pH is an essential environment for their function and the required acidic pH is produced by a V-ATPase. Interestingly, the hyperpigmentation in CALM is characterized by increased melanin in melanocytes and basal keratinocytes [42]. In mammals, mature melanosomes are transported from melanocytes to keratinocytes [43]. Furthermore, mutations in V-ATPase subunits produce pigment dilution phenotypes in *Drosophila*, zebrafish, mice and humans [44], [45]. Since V-ATPase function has been shown to be essential for melanosome biogenesis, we hypothesize that the pigmented phenotype of CALM may be a consequence of increased expression of *ATP6V0B* and an increase in the number of mature melanosomes produced in melanocytes (or heightened pigmentation) and/or transported to surrounding keratinocytes. However, the potential effect of the variant G allele of SNP rs4660761 on the expression of *ATP6V06* in melanocytes is not known and thus testing these hypotheses and the tissue-specific nature of ATP6V06 function remain interesting biological questions for the future.

The gene *DPH2* is involved with diphthamide synthesis, which is a post-translational modification of histidine residue 715 on elongation factor 2 (eEF2), a housekeeping protein involved in elongation of translation [46]. This modification is exceptional in that it occurs only on eEF2 [47]. Yeast strains lacking *Dph2* are prone to increased frequency of (−1) frameshifting by the ribosome during translation. In mice, inactivation of one copy of *Dph1* or *Dph3*, two of the five genes involved with murine diphthamide modification, increases incidence of tumor development, while inactivation of both copies of either gene is embryonically lethal. Human *DPH1* (also known as *OVCA1*, ovarian cancer-associated gene 1) inhibits the proliferation of epithelial ovarian cancer cells [48]. These observations imply that *Dph* genes and diphthamide modification of eEF2 may affect accuracy of protein synthesis in the cell, the rate of tumor incidence and other developmental processes.

We found variation in *MSH6* associated with CALM count, although these SNPs did not validate as convincingly as those in *DPH2*. However, *MSH6* deserves special note. It is a member of the DNA mismatch repair (MMR) family of genes, which ensures fidelity of DNA replication. Hereditary nonpolyposis colorectal cancer (Lynch syndrome) is caused by heterozygous germline mutations in MMR genes (including *MSH6*) [49]. Individuals with homozygous or compound heterozygous mutations in *MSH6* develop an NF1-like phenotype with multiple CALM as well as central nervous system, hematologic and gastrointestinal malignancies [50]–[55], perhaps secondary to post-zygotic mutations in *NF1* [56]. Zebrafish models of MMR deficiency also feature neurofibromas and other NF1-associated tumors [57].

This study's strengths include thorough, prospective, quantitative phenotyping of a cohort of individuals who all met diagnostic criteria for NF1. We used rigorous statistical analysis of two additional cohorts to validate findings from the discovery cohort. We acknowledge several limitations. We used LCLs as the source of tissue for our expression studies. As a proxy, LCLs are easy to obtain and culture, but there is limited overlap in blood expression profiles and other tissues [25]. We did not determine the germline mutation of *NF1* in each participant in the DISC and REP1 cohorts, given the limited genotype-phenotype correlation in the disorder. However, there were no *NF1* microdeletions in the DISC and REP1 cohorts [58], nor did we detect the 3-basepair in-frame deletion (NM_000267.3:c.2970_2972delAAT) of exon 22 (legacy exon 17), an *NF1* genotype know to affect neurofibroma number [3], [58]. In the REP2 cohort there were three individuals with an *NF1* microdeletion, although this is not known to affect CALM count. *NF1* mosaicism is frequently invoked to explain milder disease presentations, but it is difficult to prove or disprove its existence in an individual. In the DISC cohort, 77 (58%) individuals presented *de novo* NF1, and were more likely to be mosaics or of unknown inheritance. *NF1* mosaicism is approximately 10 times less common than the prevalence of germline *NF1* mutations itself [59]. We conservatively estimate that 10% of the *de novo*/unknown inheritance group (approximately 8 individuals) in our study of 132 individuals (6%) may be NF1 mosaic. This modest percentage is unlikely to influence our study results.

Identifying common genetic modifiers of monogenic disorders is akin to the detecting common genetic variation influencing traditional complex traits [60]: both are difficult to study, prone to small effect sizes and dependent on the selection of the proper phenotype [61]. Efforts to identify genetic modifiers of tumor burden or severity in the NF1 mouse model yielded alleles with modest effects but required sizable, complex breeding schemes [62]. The SNPs we identified were associated with CALM count, which is among the most heritable of all NF1 features [11], [13]. Tractability of phenotype is also important; CALM count is relatively easy to measure and is established by early childhood, although the lesions may fade with age. Our work is proof that genetic modifiers of the NF1 phenotype can be identified. Efforts to identify variants influencing time-dependent phenotypes (*e.g.* dermal neurofibroma burden) will require careful phenotyping and large, collaborative efforts.

## Materials and Methods

### Patient recruitment

The DISC and REP1 cohorts were comprised of adults meeting the consensus criteria for the diagnosis of NF1 [63], [64] who were willing to travel to the NIH Clinical Center in Bethesda, Maryland and who had both living biological parents willing to donate a blood sample. The parents did not need to be affected with NF1. Exclusion criteria for probands included: 1) any past or present history of radiation therapy, chemotherapy or biologic agents that might be expected to alter the natural history of neurofibroma growth, 2) any history of surgery to remove multiple neurofibromas or spinal neurofibromas, 3) cognitive delay that would preclude sedation to obtain an MRI, 4) presence or suspected presence of surgical hardware (*e.g.*, Harrington rods) or metallic objects that would preclude MRI imaging and 5) inability or unwillingness to tolerate an extended (one hour or more) MRI protocol. Study participants were recruited via a variety of means (*e.g.*, Google advertising, letters to NF1 clinics) from throughout the United States. Travel and lodging costs were covered by the protocol. Lymphoblastoid cell lines (LCLs) from the first 79 participants (“EXPR”) were used in the gene expression screen to identify putative modifiers. For tests of association of variants in putative modifiers identified in the EXPR screen, 99 participants were used as a discovery cohort (DISC) where 70 samples of the EXPR cohort were included in the DISC sample. An additional independent 33 and 81 participants were used as validation cohorts (REP1 and REP2, respectively). This study was approved by the National Human Genome Research Institute and National Cancer Institute institutional review boards and all participants provided written, informed consent.

### NF1 quantitative phenotyping and biospecimen collection: DISC and REP1 cohorts

We sought to quantify the NF1 phenotype in a comprehensive two-day visit to the NIH Clinical Center. A single observer (DRS) performed a history and physical exam (with measurements), Wood's lamp exam, slit-lamp exam, and collected photographs of the skin. NF1-specific abnormalities were noted (*e.g.*, presence/absence of intertriginous freckling, bony abnormalities, dysmorphic features) and a clinical assessment of the probability of mosaic NF1 was made. Whole-body cutaneous neurofibroma burden (lesions projecting above the skin) was estimated within a set of ranges (0, 1–10, 11–50, 51–100, 101–500, 500+). In addition, a paper frame with a 100 cm^2^ cut-out at the center was placed on the mid-back, abdomen and left thigh of each participant and a photograph was taken. Within the 100 cm^2^, all protruding cutaneous neurofibromas greater than 2 mm were counted, marked with water-soluble ink and re-photographed. The number of cherry hemangiomas, an under-recognized feature associated with NF1 [65], [66], was also counted within each frame at the three different sites. The number, size and distribution of CALM and other dermatologic abnormalities were counted, measured and mapped with a Wood's lamp and ruler in a darkened room. The CALM count was defined as the total number of café-au-late spots greater than 5 mm in any dimension. A slit-lamp exam was used to enumerate and photograph Lisch nodules in the eye, as previously described [58]. From the physical exam we measured height, weight and head circumference. Growth charts specific for the NF1 population (recruited at an Italian center) were used to determine centile rankings of height and weight [67]. Centile charts for adult head circumference (adjusting for gender and height) were also used for NF1-affected individuals [68]. We obtained demographic and self-reported ethnicity data, a pedigree and associated data (parity, presence of consanguinity, age of parents at birth), subject and parental heights, an MRI of the spine and clinical photographs, and referred participants to the dental clinic at the NIH Clinical Center for a cephalogram, panograph, and intra-oral photography. All participants received genetic counseling. Blood samples for DNA extraction, RNA extraction (PaxGene tubes) and for LCL production were drawn on morning of the second day of the evaluation.

### Patient recruitment and phenotyping: REP2 cohort

Patients with NF1 were enrolled in the “Neurofibromatosis Type 1 Natural History Study” (NCT00924196), approved by the NCI Institutional Review Board. Patients or their guardians were provided written informed consent. Eligibility criteria included a clinical diagnosis of NF1 or presence of an *NF1* germline mutation. A detailed skin evaluation at the time of enrollment by a single observer (AMB) was used. The number, size and distribution of CALM >5 mm in any dimension were recorded. They were measured with a ruler and documented on a standard form utilized on the natural history study.

### Establishment and culture of LCLs for EXPR screen

All LCLs were established from peripheral white blood cells at the Lombardi Comprehensive Cancer Center, Georgetown University, using standard procedures. Cells were stored in liquid nitrogen until needed for an experiment. To minimize batch effects, 79 cell lines were thawed on the same day and seeded at initial density of 500,000 cells per mL in 12-well plates. The cultures were maintained in an incubator at 37°C with 5% CO_2_ in RPMI 1640 medium supplemented with 2 mM L-glutamine, 100 Units/mL penicillin, 100 mg/mL streptomycin and 15% heat-inactivated fetal bovine serum. The cells were fed every other day and harvested on the same day after 10 days of culturing. The cell densities in the fastest and slowest growing cultures were 1.9 and 1.1 million cells/mL on the day of harvesting, respectively. The majority of LCLs exhibited similar growth rates and were at density of 1.3 to 1.7 million cells/mL at the time of harvesting. For harvesting, the cells were transferred into 15 mL tubes, spun at 400 g for 5 min at room temperature, washed once with PBS (no Ca^++^ or Mg^++^), spun again, and the pellets were lysed in 1 mL of Trizol reagent. The lysates were stored at −80°C prior to RNA extraction. All reagents were from Life Technologies (Grand Island, NY, USA).

### RNA extraction and Illumina microarray expression profiling

For RNA isolation, Trizol cell lysates were mixed with chloroform (1/5 of lysate volume), vortexed for one minute and centrifuged in a table-top centrifuge at 13,000 rpm for 15 min at 4°C. The aqueous phase containing RNA was mixed with an equal volume of 70% ethanol and immediately loaded onto RNeasy mini columns (Qiagen, Valencia, CA, USA), with subsequent steps performed as per the manufacturer's protocol. The RNA quality was estimated on a 2100 Bioanalyzer, RNA 6000 Nano Chips (Agilent, Santa Clara, CA, USA). Samples with RNA integrity number (RIN) of 8.0 and above were used for further analysis. For microarray analysis of RNA, all reagents, consumables, lab-ware, instruments, and software were obtained from Illumina, Inc (San Diego, CA, USA) unless otherwise indicated. RNA amplification/labeling, microarray hybridization, and microarray washing/staining and scanning procedures were done according to the Illumina protocols without modifications. Amplified biotinylated cRNA (1.5 µg) was hybridized to HumanRef-8_v2 Sentrix BeadChips. Samples were hybridized to microarrays at 55°C for 16–17 hours. Microarrays were washed to remove non-specifically bound cRNA, stained with 1 mg/mL Streptavidin-Cy3 (GE Healthcare, Piscataway, NJ, USA), dried, and scanned in an Illumina BeadStation 500 scanner. Image acquisition and initial image analysis were done with Illumina BeadScan and BeadStudio applications. Raw expression data were quintile normalized, background subtracted, floored to remove negative values and transformed by calculating logarithm, base 2, for each value (for better approximation to a normal distribution).

### Regression analysis and data filtering

Simple linear regression analyses between specific NF1 quantitative phenotypes (height, head circumference, total number of CALM count, cutaneous neurofibroma burden, Lisch nodule count and cherry hemangioma count) and expression values obtained for each individual in the EXPR set (Table 1) were performed for each of the 22,177 transcripts on the microarray. The FDR calculation procedure was applied to correct for multiple testing [69]. All phenotype-transcript regression pairs with an FDR below 0.3 were considered significant. The output was further filtered by subtracting phenotype-transcript pairs with expression level of transcripts below 6 (mean log_2_), expression range (difference between maximum and minimum expression) below 2 and considering biological significance of the candidate genes. In some cases, genes with an expression level below 6 and an expression range below 2 were still considered for validation because of their biological importance.

### Quantitative PCR verification of significant transcripts

Filtered transcripts with putative phenotype/expression correlates (Table S1: “Set of 80”) were investigated for outliers by generating scatter plots of quantitative phenotype *vs.* transcript expression values. Twenty-one select transcripts (Table S1: “Set of 21”) plus an endogenous control (*GAPDH*) were interrogated by qPCR in all 79 samples that were analyzed on microarrays on 384-well microfluidic cards (Applied Biosystems, Carlsbad, CA, USA). The microfluidic cards were processed and analyzed per the manufacturer's instructions without modifications. Relative expression of each gene was calculated using the standard “double delta Ct” method, per the manufacturer's protocol. Simple linear regression analysis of the qPCR expression values and corresponding quantitative phenotypes was performed as described above. For a given transcript, correlation of qPCR expression with phenotype with a nominal *p* value less than 0.05 was considered significant.

### Candidate gene sequencing in the discovery (DISC) sample set

Coding and limited evolutionarily conserved non-coding sequences of *MSH6*, *MSH2*, *MLH1*, *MED21* and *DPH2* were sequenced from germline DNA using the dideoxynucleotide chain termination method (Sanger). The genes *MSH6* and *MED21* were sequenced because of prior validation by qPCR. We included *MSH2* and *MLH1* because the protein products of these genes are known to associate with *MSH6* in functional MMR complexes. Despite not being verified by qPCR, *DPH2* was included because of its biological significance. The concentration of genomic DNA (gDNA) used in sequencing was determined using a DyNA Quant 200 fluorometer (Hoefer, Holliston, MA USA) and dsDNA-specific Hoechst Dye 22358 according to the manufacturer's protocol. The gDNA sample was then tested for functionality in PCR reactions with positive and negative control primers:

Pos_For: TGTAAAACGACGGCCAGTATCCCACTGTTAGGAGAACTGC

Pos_Rev: CAGGAAACAGCTATGACCGGTCAGGAAAGGGACACAGATA

Negative control primers are the forward and reverse sequencing primers to lac-Z of M13:

M13_For: TGTAAAACGACGGCCAGT

M13_Rev: CAGGAAACAGCTATGACC

To each gDNA sample, a trace amount of a plasmid with a unique non-human insert was added to serve as a biological barcode; the identifying inserts were amplified and checked using the universal sequencing primers above. The gDNAs were diluted to a working concentration of 2.5 ng/µL. To amplify gDNA, primers were obtained from Eurofins MWG Operon (Huntsville, AL, USA) in individual tubes and reconstituted to 100 µM in 10 mM TRIS, pH 8.0, 0.1 mM EDTA. The primers pairs were tested at a concentration of 0.16 µM each in 10 µL PCR reactions containing KAPA 2G Fast HS ReadyMix PCR Kit (KAPA Biosystems, Woburn, MA, USA) and 5 ng of control human DNA (Coriell Institute, Camden, NJ, USA). Cycling conditions: 1) activate enzyme at 95°C for 3 min, 2) 40 cycles at 95°C for 10 sec, 60°C for 10 sec and then 72°C for 30 sec and, 3) hold at 10°C. A 5 µL aliquot of the PCR reaction was examined by agarose gel to assess multiple or missing bands. The PCR products were then diluted to 0.4 ng/µL and sequenced in 6 µL reactions using M13 universal forward and reverse primers and BDT version 3.1 (Applied Biosystems) using standard ABI protocols. The reactions were then analyzed on 3730 DNA Sequencers (Applied Biosystems). The sequence traces were individually inspected for quality. Primer pairs that did not lead to high-quality traces were retested using one additional control DNA. Primers failing both rounds were redesigned. PCR amplification of amplimers was performed in 10 µL reactions in 384-well plates, as described above. Prior to sequencing, the PCR products were diluted to 0.4 ng/µL. Sequencing was performed on an Applied Biosystem 3730 Sequencer using BigDye Terminator version 3.1. Three µL of diluted PCR products were used in sequencing reaction volumes of 6 µL. Sequencing primer sequences are as above. Reaction cleanup was accomplished through alcohol precipitation. Reaction precipitates are dissolved in 10 µL water immediately before sequencing. All genomic coordinates reference the hg18 (March 2006) build.

### Candidate SNP genotyping in the REP1 sample set

#### 1) PCR and sequencing

For genotyping *MSH6* SNP rs1800934 in an independent set of germline DNA from 33 samples, a 632 bp amplimer was generated from gDNA using: 5′-GTAGTCCGCCCACCTAAGC (forward) and 5′-CCCTAGCTCTCTACTTCTTACCAAAA (reverse). The primers were appended with universal sequences at their 5′-ends: 5′-TGTAAAACGACGGCCAGT (forward), 5′-CAGGAAACAGCTATGACC (reverse). PCR and sequencing was performed as described above.

#### 2) Illumina Human OmniQuad-1M SNP-arrays

For genotyping *DPH2* SNPs rs4660761 and rs7161, we used genotyping calls obtained from Illumina Human OmniQuad-1M SNP-arrays. SNP-array analysis was done according to the manufacturer's protocol using GenomeStudio (v. 2010.2) software (Illumina).

### Candidate SNP genotyping in the REP2 sample set

A targeted, multiplex PCR primer panel was designed using the custom Ion Ampliseq Designer v3.0 (Thermo Fisher Scientific, Life Technologies, Carlsbad, CA, USA). The primer panel covered 11 kb of sequence that includes the specific variants of interest in the *MSH6* and *DPH2* loci. Each site was 100% covered in the design. Average amplicon size was 225 bp. Sample DNA was amplified using this custom Ampliseq primer pool, and libraries were prepared following the manufacturer's Ion Ampliseq Library Preparation protocol (Life Technologies, Carlsbad, CA, USA). Individual samples were barcoded, pooled, templated, and sequenced on the Ion Torrent PGM Sequencer using the Ion PGM Template OT2 200 and Ion PGM Sequencing 200v2 kits per manufacturer's instructions. Mean read length after sequencing was 159 bp.

### Statistical analysis of SNPs putatively associated with café-au-lait macule count in the DISC sample set

#### Data preparation

The CALM count trait was used both as the untransformed (unt) and as the log-transformed trait (log) by log_10_(x+10). Single nucleotide variants (SNVs) from the five selected candidate genes (*MSH6*, *MSH2*, *MLH1*, *MED21* and *DPH2*) were filtered based on the with the following: 1) if the polyphred score for an SNV was less than 99, the genotype was deemed “missing”; 2) if a sample had greater than 30% of its SNVs “missing” then the sample was excluded; 3) if an SNV had a greater than 20% “missing” rate in all of the samples, the SNV was excluded; and 4) all monomorphic SNVs were excluded. In those cases in which family data were available, SNVs were checked for Mendelian segregation with PedCheck [70]. Hardy-Weinberg equilibrium (HWE) proportions were tested with PEDSTATS [71] on 70 unrelated individuals. Two SNVs not in HWE (*P* value<0.03) were flagged but retained for analysis since removing SNVs not in HWE in highly-ascertained samples may remove causative SNVs. Self-reported European-American samples were included for further analyses. After filtering, there were a total of 91 individuals with 118 SNVs.

#### Simple linear regression and tiled regression

Simple linear regression was performed on each SNV after adjusting for age and sex. “Tiled Regression” as implemented in TRAP (Tiled Regression Analysis Package) [72] was used to identify the set of independent significant variants considering all the SNVs in the sample that affected the number of CALM after pre-adjusting for age and sex. Both simple linear regression and tiled regression were performed under two models, additive and dominant for both the untransformed and log-transformed traits. Briefly, in tiled regression, the genome is divided into independent segments based on predefined regions called tiles. In this study, tiles were defined by hotspot-based regions, delimited by the location of recombination hotspots in Human Genome Sequence build 36, yielding 5 independent tiles. Each tile was first analyzed with multiple linear regression of the trait on all SNVs in the tile and by simple linear regression of the trait on each SNV separately. Only those tiles for which the overall multiple linear regression showed a significant relationship to trait variation at the level of 0.2, or in which simple linear regression on any single SNV was significant at the level of 0.05, were retained for subsequent analyses. A forward stepwise regression with backward look then was performed within each tile to select the important individual independent SNVs identified with a critical level of 0.05 for entry and for retention in the model. Thereafter, the SNVs retained from each tile were combined across tiles for higher order stepwise regressions at chromosome and then whole genome levels using the same critical values. The end result was a multiple linear regression model that included the set of variants independently contributing to trait variation.

#### Variant coding

Genotypes for 118 SVs, including both 58 common and 60 rare variants (defined by minor allele frequency <0.05), were used as provided (un-collapsed), and with the rare variants (RVs) collapsed. Several coding schemes were considered: 1) the number of minor alleles (add) for each common variant (CV) and for each un-collapsed RV, 2) the presence or absence of the minor allele (dom) for each SNV and for each un-collapsed RV, 3) the number of minor alleles (add) for each CV and, with a new collapsed variant that was coded as proportion of a minor allele at any RV; in other words, collapsing multiple RVs into a single region-wide variant within hotspot-based region definition.

### Validation of SNVs significant in the DISC sample set associated with café-au-lait macule count in independent sets REP1 and REP2

To confirm the association of SNVs in the DISC sample set with CALM count, we genotyped the variants in germline DNA in an independent set of 33 samples (REP1) and an additional independent set of 81 samples (REP2). Since none of the significant SNVs in the DISC set were significantly associated with CALM count by simple linear regression at the level of 0.05 based on 29 and 62 European-American samples in REP1 and REP2, respectively (Table 2), we performed additional analyses. In the meta-analysis, p-values of these three (DISC, REP1, REP2) datasets were combined using Liptak's method [73] by weighting each p-value by its square root of the sample size (Table 2). In the mega-analysis, three (DISC, REP1, REP2) datasets including 180 samples were combined and simple linear regression was performed on each SNV by adjusting for age, sex and each dataset (Table 2). Tiled regression was not performed in the replication study since the method requires genotyping all variants, not just markers of interest. We did not attempt to validate rare variants due to limited size of the additional set.

#### Bioinformatic exploration of *DPH2* and *ATPV0B* SNP function

To explore whether SNPs rs4660761 and rs7161 might have potential regulatory functions in skin cells (including melanocytes), we used custom tracks on the UCSC Genome browser (http://genome.ucsc.edu) to screen Roadmap and ENCODE data containing the implicated SNP regions for evidence for regulatory relevance [74]–[76], such as overlapping with chromatin marks and interactions, CpG-site methylation and transcription factor binding motifs. We also used the online tools HaploReg (http://www.broadinstitute.org/mammals/haploreg/haploreg.php) and RegulomeDB (http://regulome.stanford.edu) as a complementary analysis and to confirm the location of each SNP in relation to annotated protein-coding genes and/or non-coding RNA (ncRNA) genes.

The content of this publication does not necessarily reflect the views or policies of the Department of Health and Human Services, nor does mention of trade names, commercial products or organizations imply endorsement by the U.S. Government.

## Supporting Information

Figure S1 (**A–H**) Scatter plots of gene expression (*MSH6*, *DPH2*, *MED21*, *NMT2*, *TMEM109*, *FHL2*, *PREB*, *RAB11FIP1*) against select NF1 phenotypes.
(PPTX)

Table S1 Genes significantly associated with clinical NF1 phenotypes. Filtered transcripts with putative phenotype/expression correlates (“Set of 80”); transcripts for qPCR verification (“Set of 21”) and qPCR-verified transcripts (“Verified 7”) for each of the six quantitative traits. qPCR-verified genes are shown in blue font.
(XLSX)

Table S2 Nominal p value of correlation between gene expression (by microarray or qPCR) and NF1 quantitative trait for 7 transcripts significantly associated with phenotype severity. Traits include café-au-lait macule (CALM) count (total number), Lisch nodules (LN) count (total number) and height (centile ranking for NF1 population as per Clementi et al. 1999 growth charts). All genes but TMEM109 were significant in all NF1-affecteds combined and in one of the gender subgroups; TMEM109 was significant in NF1-affected males only. We observed this gender-specific pattern of association in the microarray results as well (Figure S1).
(XLSX)

Table S3 Significance of association of rare SNVs collapsed with CALM count by simple linear regression adjusting for age and sex using self-reported European-American samples
(DOC)

Table S4 Location and regulatory annotation of SNPs associated with CALM count.
(XLS)
