# Supplementary material for: Genetic Modifiers of Neurofibromatosis Type 1-Associated Café-au-Lait Macule Count Identified Using Multi-platform Analysis
Source: PLoS Genet. 2014 Oct 16;10(10):e1004575. doi: 10.1371/journal.pgen.1004575 (PMC4199479; doi:10.1371/journal.pgen.1004575)
Supplement: Figure S1 — (A–H) Scatter plots of gene expression (MSH6, DPH2, MED21, NMT2, TMEM109, FHL2, PREB, RAB11FIP1) against select NF1 phenotypes. (PPTX) [file pgen.1004575.s001.pptx]

## Slide 1
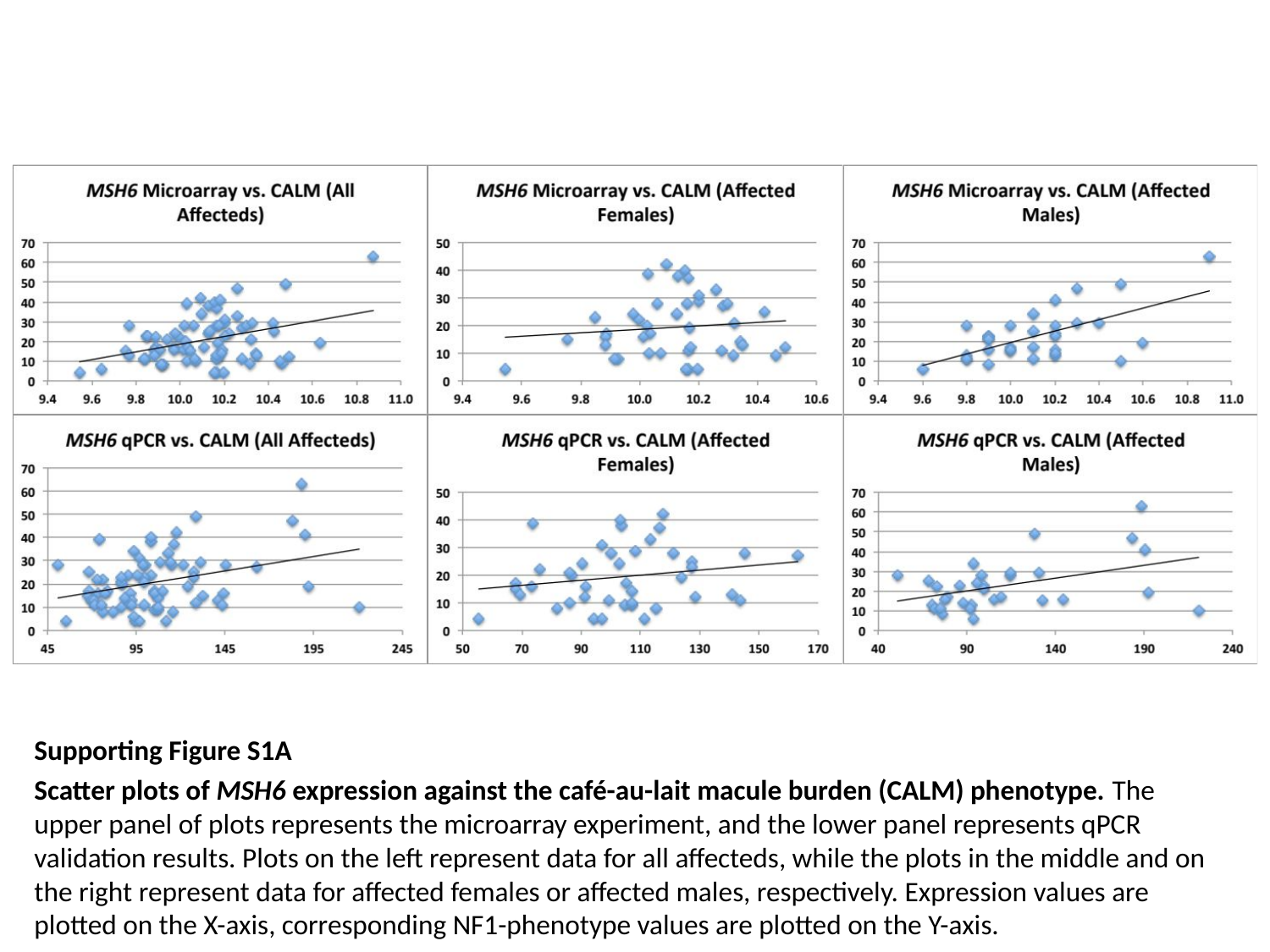

Supporting Figure S1A
Scatter plots of MSH6 expression against the café-au-lait macule burden (CALM) phenotype. The upper panel of plots represents the microarray experiment, and the lower panel represents qPCR validation results. Plots on the left represent data for all affecteds, while the plots in the middle and on the right represent data for affected females or affected males, respectively. Expression values are plotted on the X-axis, corresponding NF1-phenotype values are plotted on the Y-axis.

## Slide 2
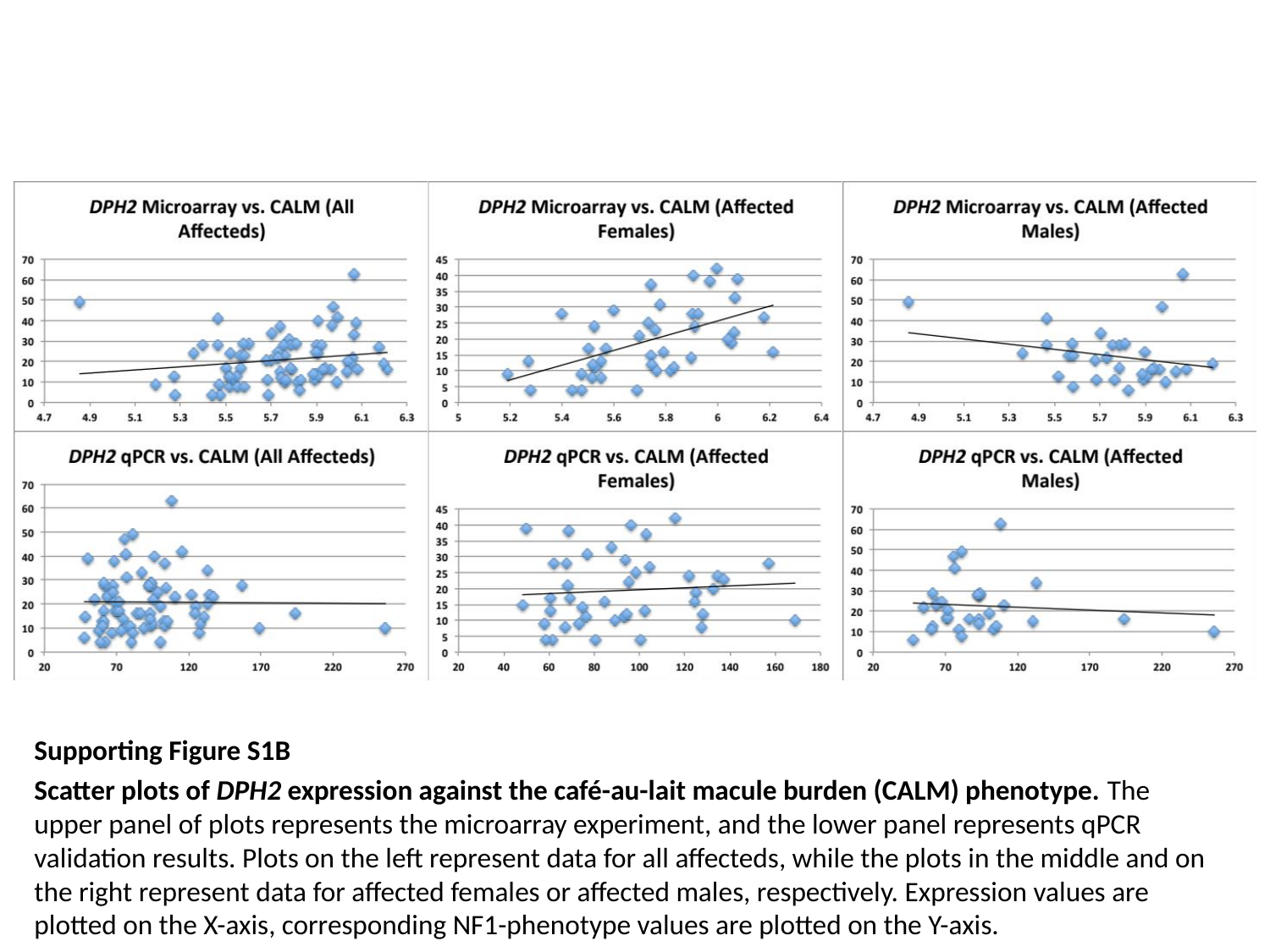

Supporting Figure S1B
Scatter plots of DPH2 expression against the café-au-lait macule burden (CALM) phenotype. The upper panel of plots represents the microarray experiment, and the lower panel represents qPCR validation results. Plots on the left represent data for all affecteds, while the plots in the middle and on the right represent data for affected females or affected males, respectively. Expression values are plotted on the X-axis, corresponding NF1-phenotype values are plotted on the Y-axis.

## Slide 3
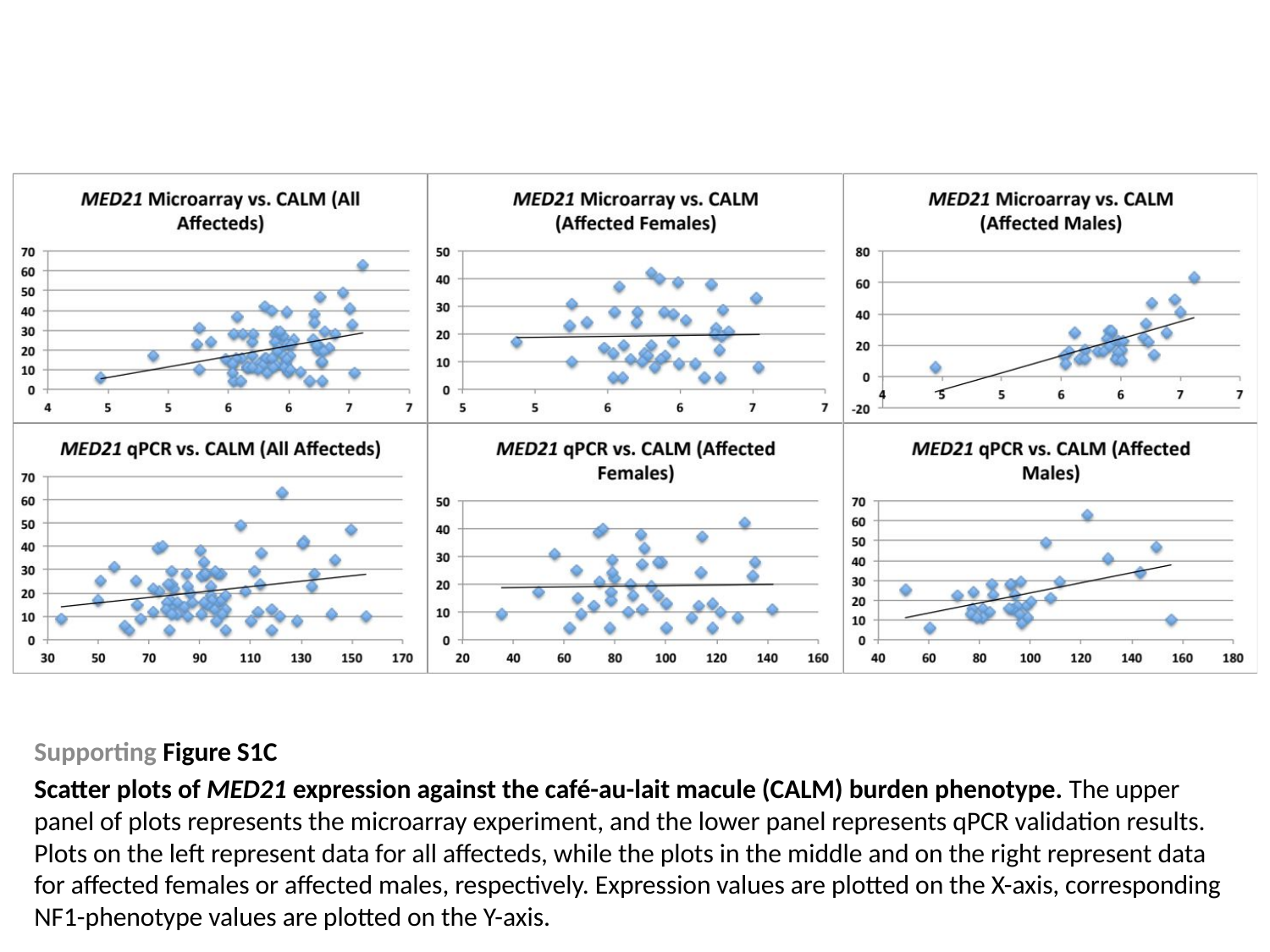

Supporting Figure S1C
Scatter plots of MED21 expression against the café-au-lait macule (CALM) burden phenotype. The upper panel of plots represents the microarray experiment, and the lower panel represents qPCR validation results. Plots on the left represent data for all affecteds, while the plots in the middle and on the right represent data for affected females or affected males, respectively. Expression values are plotted on the X-axis, corresponding NF1-phenotype values are plotted on the Y-axis.

## Slide 4
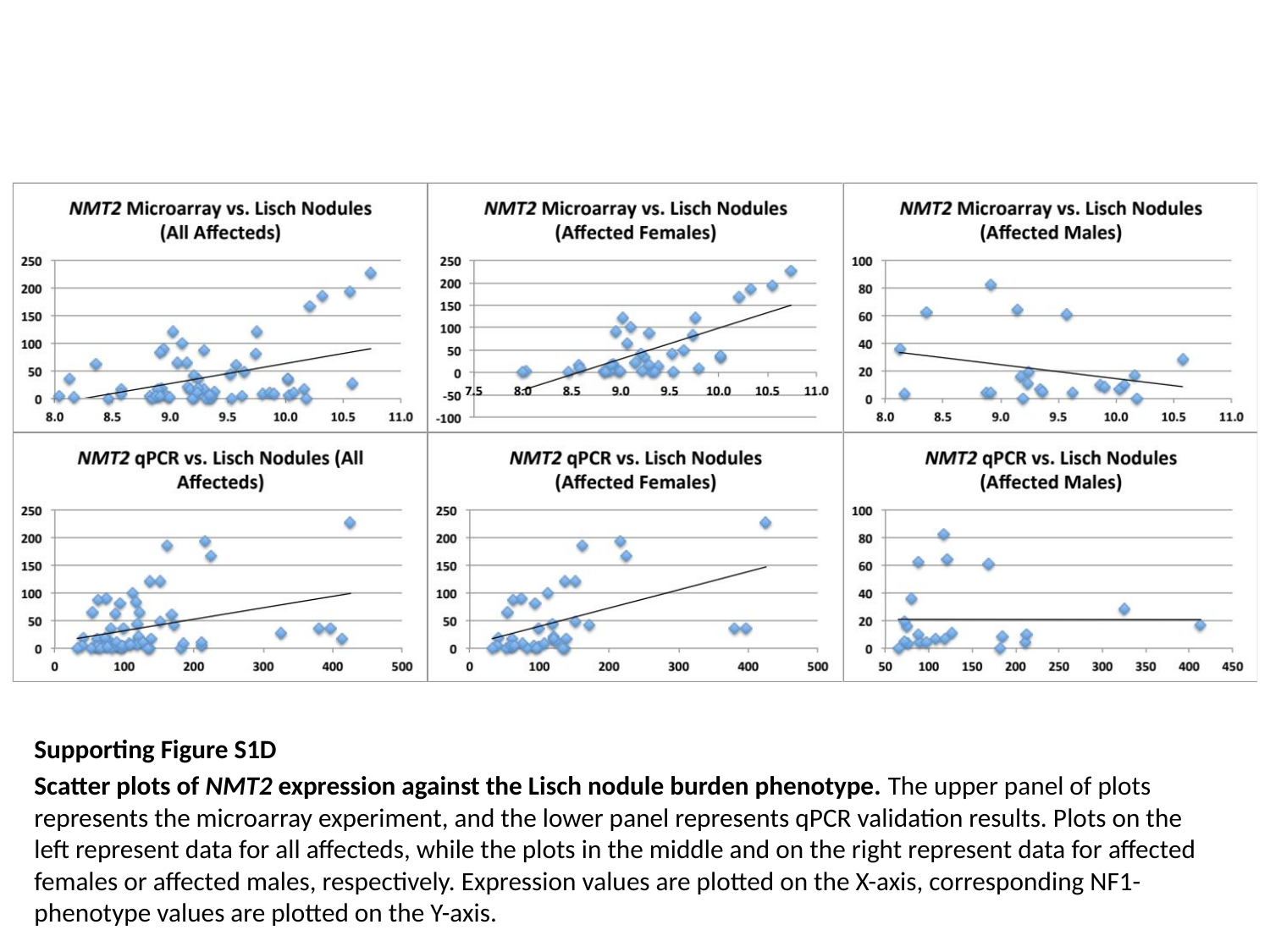

Supporting Figure S1D
Scatter plots of NMT2 expression against the Lisch nodule burden phenotype. The upper panel of plots represents the microarray experiment, and the lower panel represents qPCR validation results. Plots on the left represent data for all affecteds, while the plots in the middle and on the right represent data for affected females or affected males, respectively. Expression values are plotted on the X-axis, corresponding NF1-phenotype values are plotted on the Y-axis.

## Slide 5
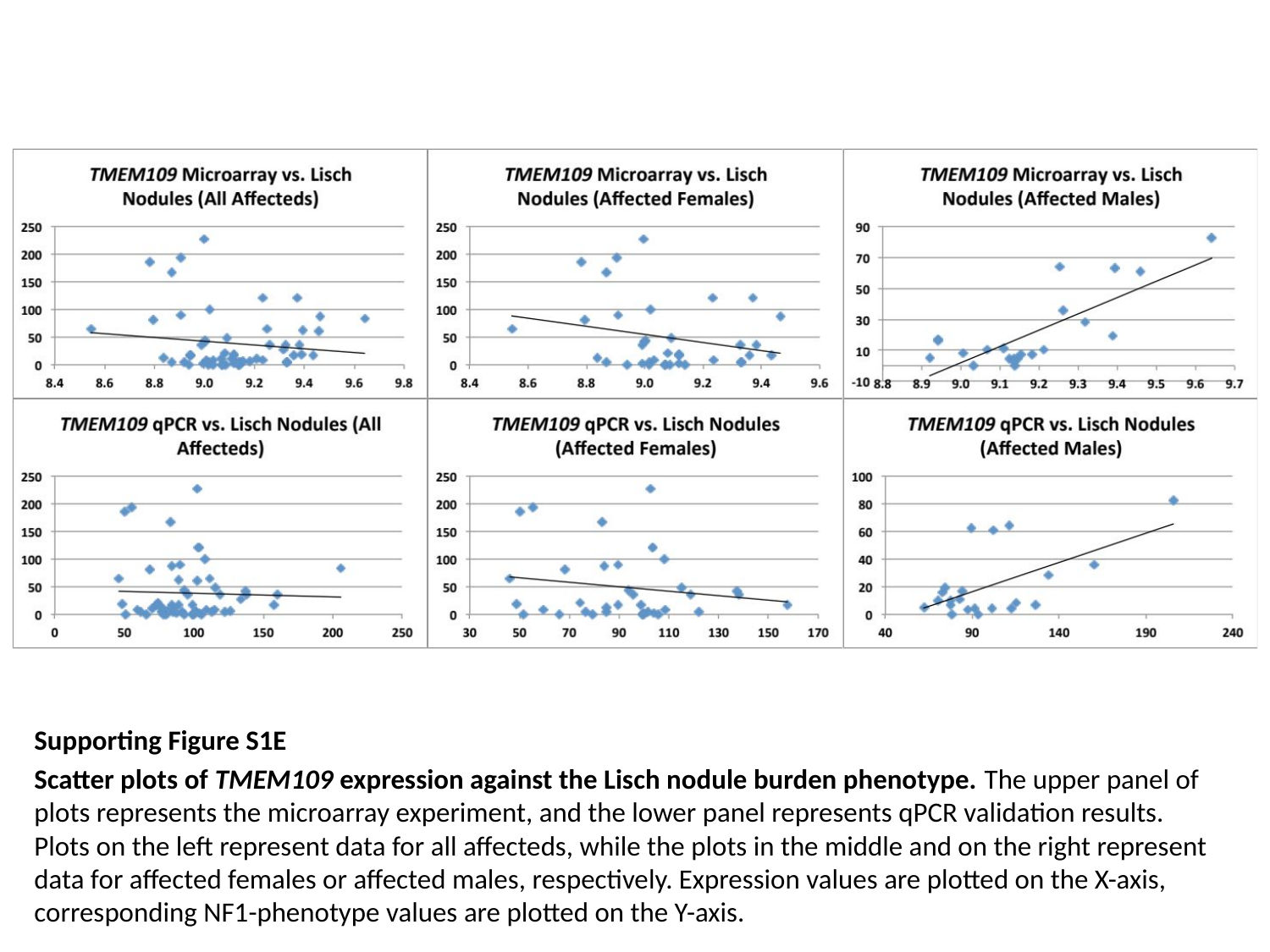

Supporting Figure S1E
Scatter plots of TMEM109 expression against the Lisch nodule burden phenotype. The upper panel of plots represents the microarray experiment, and the lower panel represents qPCR validation results. Plots on the left represent data for all affecteds, while the plots in the middle and on the right represent data for affected females or affected males, respectively. Expression values are plotted on the X-axis, corresponding NF1-phenotype values are plotted on the Y-axis.

## Slide 6
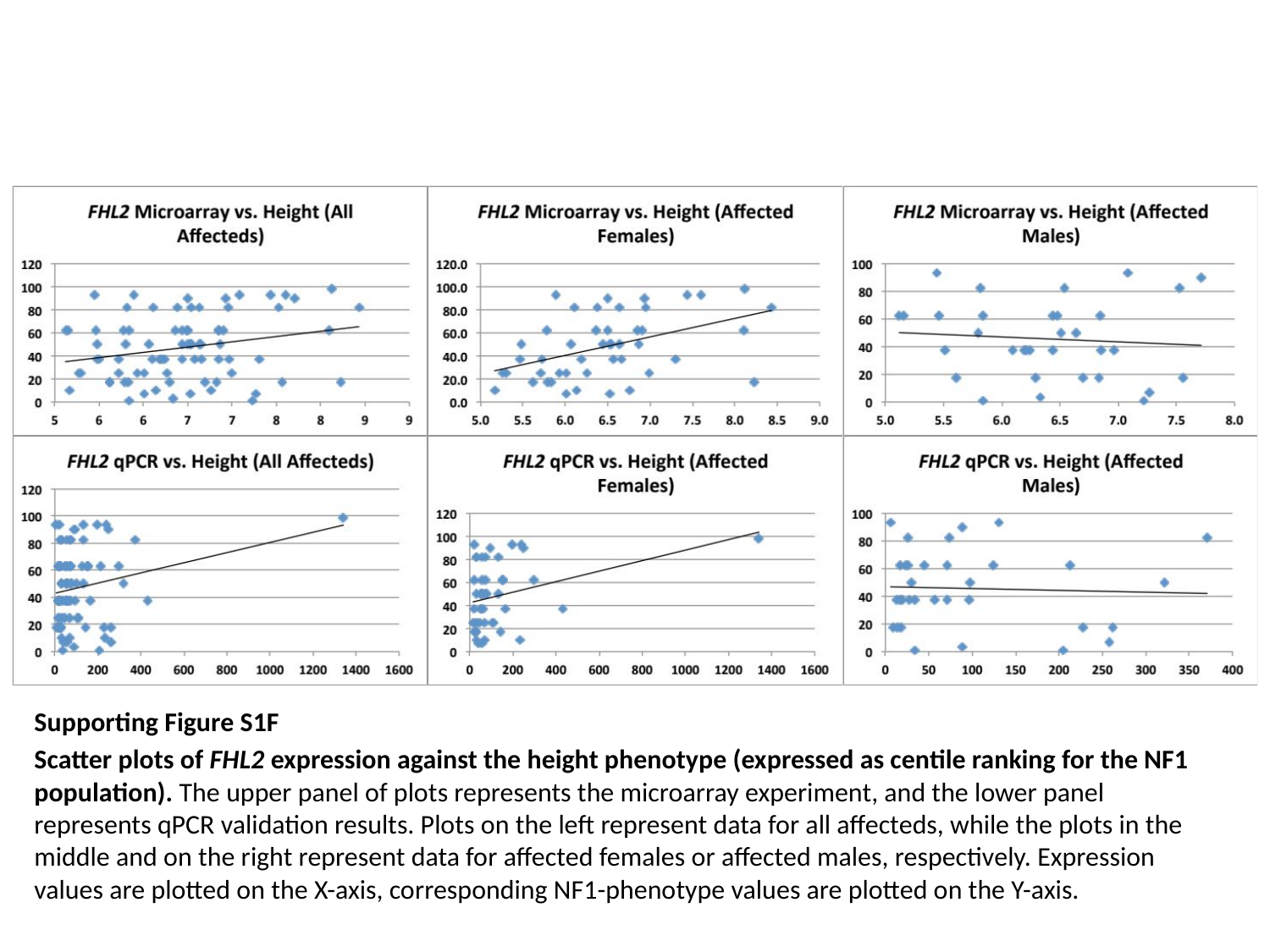

Supporting Figure S1F
Scatter plots of FHL2 expression against the height phenotype (expressed as centile ranking for the NF1 population). The upper panel of plots represents the microarray experiment, and the lower panel represents qPCR validation results. Plots on the left represent data for all affecteds, while the plots in the middle and on the right represent data for affected females or affected males, respectively. Expression values are plotted on the X-axis, corresponding NF1-phenotype values are plotted on the Y-axis.

## Slide 7
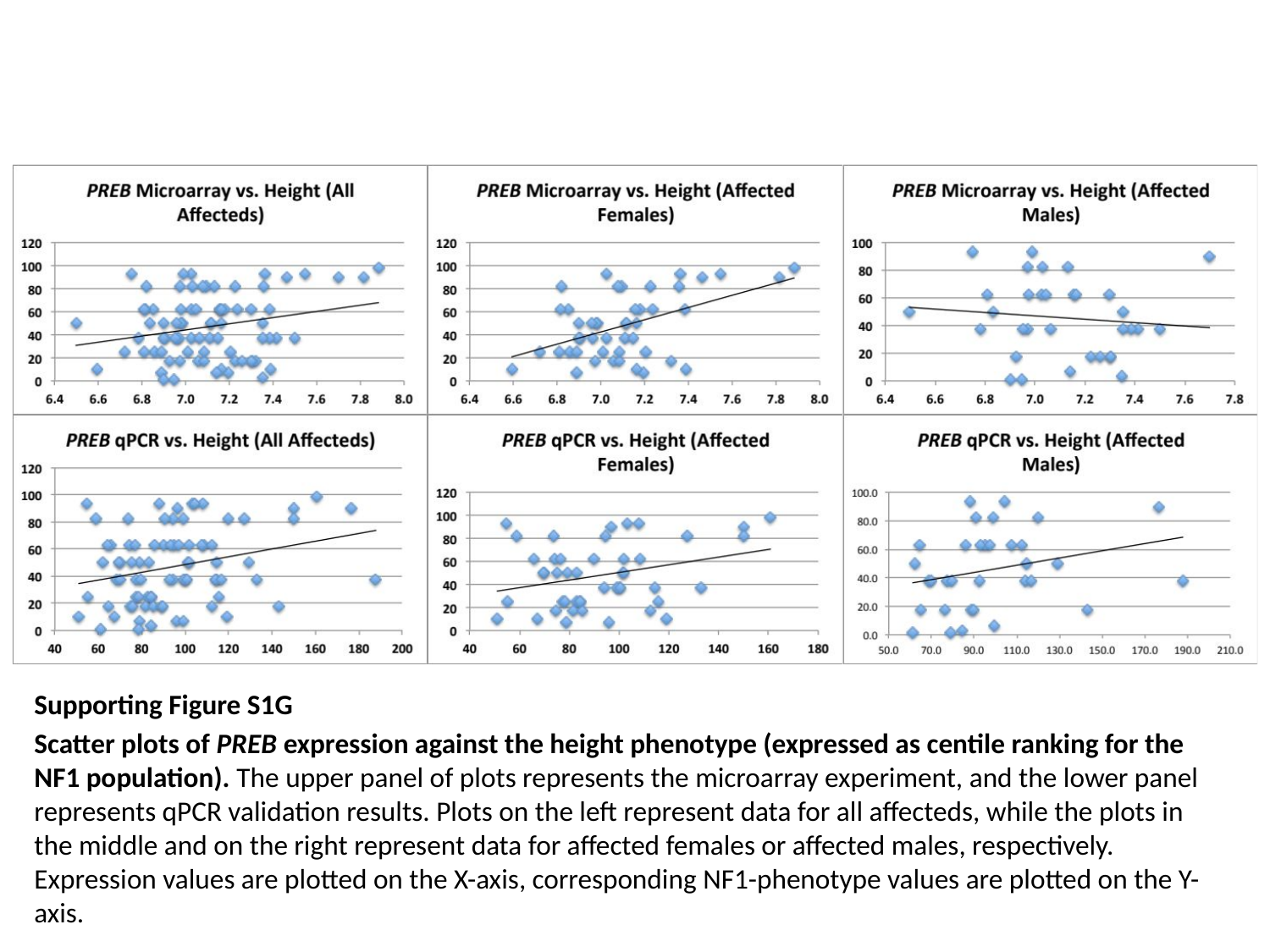

Supporting Figure S1G
Scatter plots of PREB expression against the height phenotype (expressed as centile ranking for the NF1 population). The upper panel of plots represents the microarray experiment, and the lower panel represents qPCR validation results. Plots on the left represent data for all affecteds, while the plots in the middle and on the right represent data for affected females or affected males, respectively. Expression values are plotted on the X-axis, corresponding NF1-phenotype values are plotted on the Y-axis.

## Slide 8
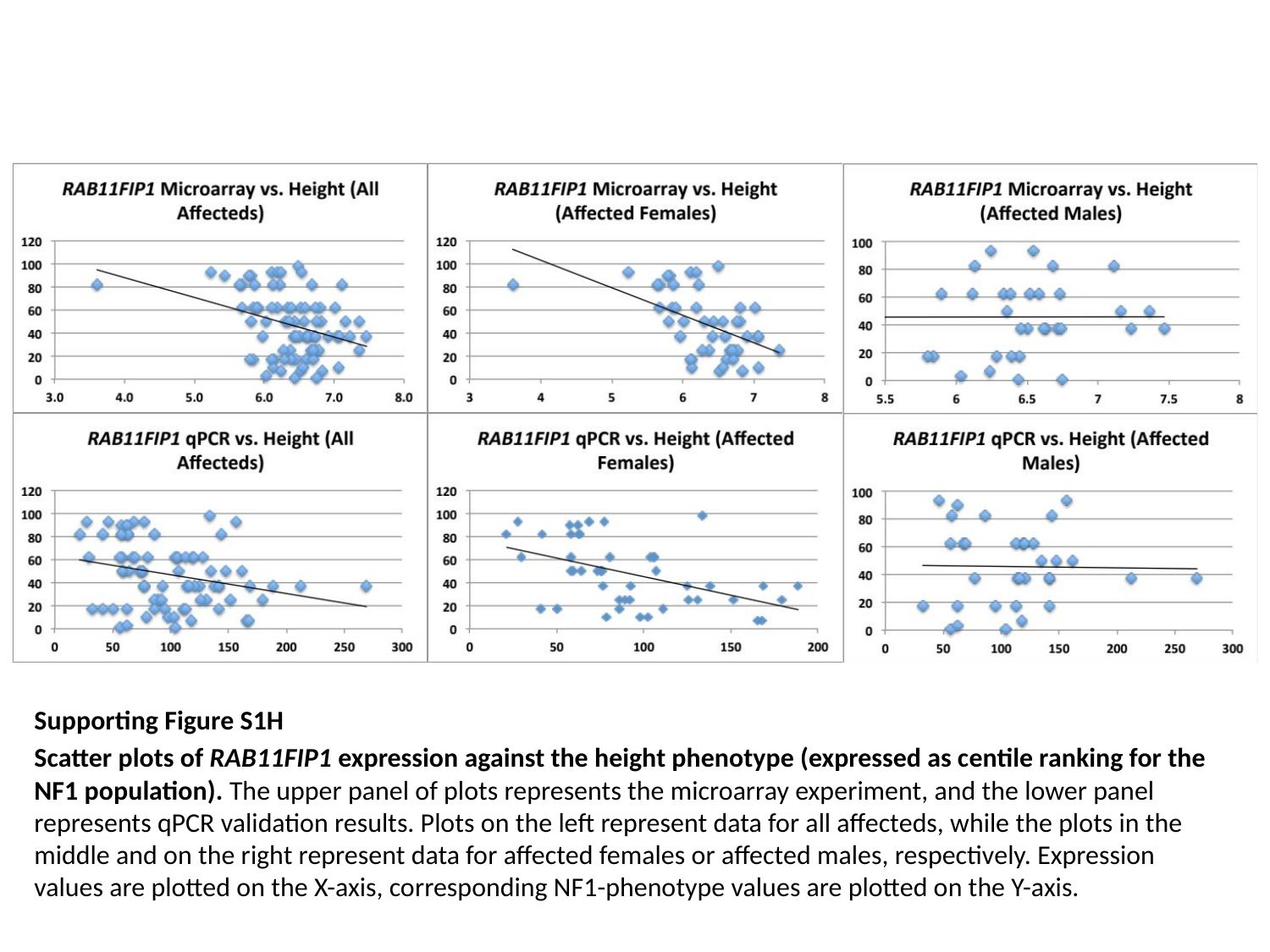

Supporting Figure S1H
Scatter plots of RAB11FIP1 expression against the height phenotype (expressed as centile ranking for the NF1 population). The upper panel of plots represents the microarray experiment, and the lower panel represents qPCR validation results. Plots on the left represent data for all affecteds, while the plots in the middle and on the right represent data for affected females or affected males, respectively. Expression values are plotted on the X-axis, corresponding NF1-phenotype values are plotted on the Y-axis.
