# Supplementary material for: Genetic Modifiers of Neurofibromatosis Type 1-Associated Café-au-Lait Macule Count Identified Using Multi-platform Analysis
Source: PLoS Genet. 2014 Oct 16;10(10):e1004575. doi: 10.1371/journal.pgen.1004575 (PMC4199479; doi:10.1371/journal.pgen.1004575)
Supplement: Table S3 — Significance of association of rare SNVs collapsed with CALM count by simple linear regression adjusting for age and sex using self-reported European-American samples (DOC) [file pgen.1004575.s004.doc]

**Supporting Table S3. Significance of association of rare SNVs collapsed with CALM count by simple linear regression** adjusting for age and sex using self-reported European-American samples

| Position (hg18)  (all *MSH6* locus) | Minor Allele Frequency from DISC | Model^a^ | | DISC (n=89) | |
| --- | --- | --- | --- | --- | --- |
|  |  |  |  | Beta (s.e.) | p-value^b^ |
| chr2; 47,863,532 bp | 0.012 | SNPs with MAF <0.05 were collapsed in hotspot-based regions coded by the proportion of the minor allele | unt | -48.627 (25.26) | 0.058 |
| chr2; 47,863,949 bp | 0.012 |  |  |  |  |
| chr2; 47,871,779 bp | 0.013 |  |  |  |  |
| chr2; 47,871,984 bp | 0.012 |  |  |  |  |
| chr2; 47,879,066 bp | 0.013 |  |  |  |  |
| chr2; 47,880,898 bp | 0.011 |  |  |  |  |
| chr2; 47,881,841 bp | 0.012 |  | log | -0.693 (0.335) | 0.042 * |
| chr2; 47,883,966 bp | 0.024 |  |  |  |  |
| chr2; 47,884,136 bp | 0.023 |  |  |  |  |
| chr2; 47,886,516 bp | 0.011 |  |  |  |  |
| chr2; 47,887,588 bp | 0.012 |  |  |  |  |
| chr2; 47,887,783 bp | 0.024 |  |  |  |  |

Note: a) unt: untransformed CALM count, log: log-transformed CALM count. b) *: p-value $\leq$ 0.05
